# Olive Oil in Scarlet Fever

**Published:** 1889-11

**Authors:** 


### OLIVE OIL IN SCARLET FEVER.

Dr. Hutchinson, in the *American Magazine*, says:

Among the many mothers who read these lines there may be one or more whose child has scarlet fever, that terrible disease that has come to be so dangerous of late years, and who will be glad to know of anything to help their baby. And this is something so simple, yet so effective, that no physician can object to its employment. It is the application to the entire body of warm sweet oil, well rubbed in. There is something curious in its immediate good effect. Almost twenty years ago I had five patients in one family with the anginose or throat variety of scarlet fever, and had them all brought into one room for convenience sake, as well as seclusion. Five little heads returned my greeting every time a visit was made, and all clamored loudly for their oil bath. No medicine was given and but little food was needed to supplement absorbed oil. And in recovery there was an absence of the usual complications, so that in my western town oil baths came to be generally used with excellent result.

"Other fats were tried, but none answered the double purpose of nutrition and skin cooler as well as plain olive oil. It is well worth trial."

